# Alloy-assisted deposition of three-dimensional arrays of atomic gold catalyst for crystal growth studies

**DOI:** 10.1038/s41467-017-02025-x

**Published:** 2017-12-08

**Authors:** Yin Fang, Yuanwen Jiang, Mathew J. Cherukara, Fengyuan Shi, Kelliann Koehler, George Freyermuth, Dieter Isheim, Badri Narayanan, Alan W. Nicholls, David N. Seidman, Subramanian K. R. S. Sankaranarayanan, Bozhi Tian

**Affiliations:** 1 Department of Chemistry, The University of Chicago, Chicago, IL 60637 USA; 2 The James Franck Institute, The University of Chicago, Chicago, IL 60637 USA; 3 The X-Ray Science Division, Argonne National Laboratory, Argonne, IL 60439 USA; 4 The Research Resources Center, University of Illinois at Chicago, Chicago, IL 60607 USA; 5 Department of Materials Science and Engineering, Northwestern University, Evanston, IL 60208 USA; 6 The Northwestern University Center for Atom-Probe Tomography (NUCAPT), Northwestern University, Evanston, IL 60208 USA; 7 The Center for Nanoscale Materials, Argonne National Laboratory, Argonne, IL 60439 USA; 8 Computation Institute, The University of Chicago, Chicago, IL 60637 USA; 9 The Institute for Biophysical Dynamics, The University of Chicago, Chicago, IL 60637 USA; 10 Present Address: Materials Science Division, Argonne National Laboratory, Argonne, IL 60439 USA

## Abstract

Large-scale assembly of individual atoms over smooth surfaces is difficult to achieve. A configuration of an atom reservoir, in which individual atoms can be readily extracted, may successfully address this challenge. In this work, we demonstrate that a liquid gold–silicon alloy established in classical vapor–liquid–solid growth can deposit ordered and three-dimensional rings of isolated gold atoms over silicon nanowire sidewalls. We perform ab initio molecular dynamics simulation and unveil a surprising single atomic gold-catalyzed chemical etching of silicon. Experimental verification of this catalytic process in silicon nanowires yields dopant-dependent, massive and ordered 3D grooves with spacing down to ~5 nm. Finally, we use these grooves as self-labeled and ex situ markers to resolve several complex silicon growths, including the formation of nodes, kinks, scale-like interfaces, and curved backbones.

## Abstract (web summary)

Parallel patterning of atoms over a large surface would represent a major advance over current serial methods of single atom manipulation. Here, the authors explore a periodic instability from liquid alloy droplets for high-throughput atom printing.

## Introduction

**Fig. 1:**
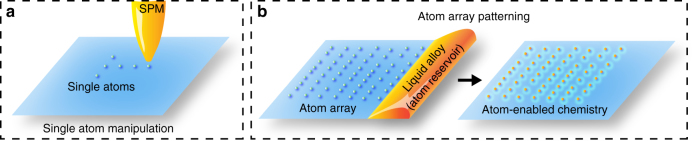
Liquid alloy may be used for atom manipulation. **a** Schematic illustrations of single atom manipulation by SPM and **b** large-scale and sequential manipulation of atom arrays with liquid alloy

Atom-by-atom manipulation by scanning probe microscope (SPM) is an effective method of assembling functional structures and devices on a solid substrate^1–3^, and has enabled numerous fundamental studies of chemical and physical processes (Fig. 1a). Despite its high precision, this technique suffers from low throughput due to its serial operation. Periodic assembly of individual atoms (e.g., aluminum and gallium) over step edges of a crystalline substrate is possible^4, 5^, especially when the bonding of adatoms at the step edges of the substrate is energetically more stable than that on the crystal terraces. However, the nanoscale features obtained this way are limited by the crystal lattice of the underlying substrates.

High throughput, sequential, and tunable printing of individual atoms over a large area has not been achieved, but if successful, it could impact fields other than quantum science^2^, surface chemistry^4, 5^, or single-molecule studies^3^. A configuration of an atom reservoir, in which individual atoms can be readily extracted, may successfully address this challenge.

The liquid alloy established in classical vapor–liquid–solid (VLS) growth could be an option for the reservoir. Indeed, in VLS growth of silicon (Si) nanowires^6–9^, many metal species such as gold (Au)^10–17^, aluminum^5, 18^, indium^19^, and tin^19^, are able to incorporate over or into Si nanowires during growth. Moreover, in a VLS process, alloy droplet instability or oscillatory motions can occur even under classical growth conditions^20–24^, and they have yielded periodic changes of crystal structure and morphology.

Here we discover an oscillatory motion of Au/Si alloy droplet, which enables a three-dimensional (3D) patterning of atomic Au over Si nanowire sidewalls. The Au atoms catalyze the etching of Si nanowires, which subsequently forms massive grooves that are used  to probe many crystal growth mechanisms.

## Results

### Concept for the parallel atom manipulation

We hypothesize that a liquid silicon-metal alloy, a reservoir of mobile and dispersed atomic species, could be explored for the parallel printing of individual atoms (Fig. 1b). We test this possibility in Au-catalyzed VLS growth of Si nanowires, because the catalysts during growth are liquid alloy droplets and they can deposit both metal nanoparticles and atoms over Si sidewalls. Besides having an atom reservoir, another key factor in controllable atom patterning is the realization of a switch that allows atom deposition only during certain time points of a sequential process. These oscillations in Au-catalyzed VLS growth can potentially be utilized for such a switch in atom printing, especially given the classical coffee-ring effect^25^ that provides information on droplet instability-induced sequential patterning of a range of nano- and microparticles.

### Discovery of atomic Au-based line patterns

**Fig. 2:**
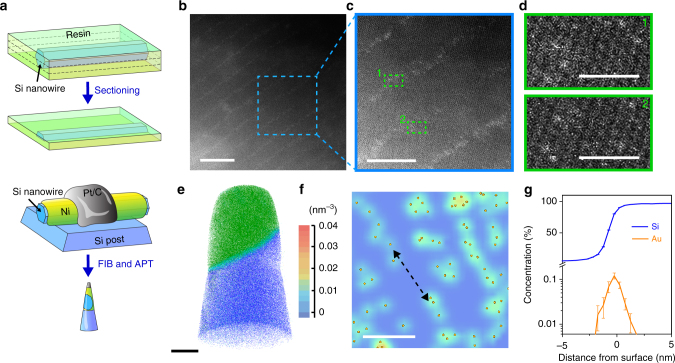
Atomic Au lines form over Si nanowire sidewalls. **a** Schematics of microtomed samples for STEM and FIB-milled samples for APT characterizations, showing the regions of interest. **b** An aberration-corrected STEM image of ordered line patterns over Si surfaces. Scale bar, 10 nm. **c** A high-resolution STEM image for a zoom-in view from **b** (region labeled with blue dashed box). Scale bar, 5 nm. **d** Isolated gold atoms images highlighted in the line regions from **c** (marked by two green dashed boxes). Scale bar, 1 nm. **e** APT analysis displays the 3D atom-by-atom chemical reconstruction of a nanowire surface region. Atomic positions are represented by blue (Si, 2.5% shown), cyan (O, 100% shown), and green (Ni, 10% shown) dots. Scale bar, 20 nm. **f** A 2D color-coded map of gold atomic density exhibits a chain-like arrangement, indicated by a black dashed arrow. Orange spheres represent gold atoms. Scale bar, 10 nm. **g** Proximity histogram concentration profile of Si and Au in the direction normal to the Si surface

Given that alloy droplet instability can occur under typical VLS conditions^20–24^, we extensively surveyed the surfaces of classical Si nanowires for the potential occurrence of atomic Au patterns over their sidewalls. We chose to focus our studies on nanowires with a diameter range of 100 nm–1 µm, as this is a critical length scale that bridges traditional nanomaterials and micron-level objects but has surprisingly received very little attention. Additionally, it is known that larger diameter in Si nanowires favors Au deposition^14, 15^. To enable high-resolution imaging of individual atoms from these relatively thick nanowires and to preserve the sample surface information, we microtomed samples for aberration-corrected scanning transmission electron microscope (STEM) imaging (thickness, ~70 nm, Fig. 2a), and used horizontally placed samples made by a focused ion beam system for laser-assisted local-electrode atom-probe tomography (APT) (Fig. 2a).

Significantly, for Si nanowires synthesized with high phosphine doping (i.e., heavy *n*-type), we consistently found ordered line patterns over Si surface with a minimum line spacing of ~5 nm (Fig. 2b and Supplementary Fig. 1a). High-resolution STEM images and energy-dispersive X-ray spectrum (EDS) indicated that the lines consisted of isolated gold atoms (Fig. 2c, d and Supplementary Fig. 1b). The fact that these gold atom lines do not align with planar defects (Fig. 2b–d and Supplementary Fig. 1c) rules out the possibility of gold trapping by a superlattice^10, 23, 24, 26^. Atomic force microscope (AFM) imaging of the Si nanowire showed smooth surfaces with a roughness less than 2 nm (Supplementary Fig. 2), ruling out another gold-trapping situation at the diameter-modulated sidewalls^22, 27, 28^.

A 3D, atom-by-atom chemical reconstruction from a nanowire surface region (Fig. 2e, green, Ni; cyan, O; and blue, Si) revealed the presence of mostly isolated Au atoms (Fig. 2f, orange spheres; Supplementary Fig. 3), with a local peak volume concentration of 0.01–0.03 atoms nm^−3^, determined at a concentration sampling grid voxel size of 0.6 × 0.6 × 1.5 nm. Compared to STEM imaging, the APT chemical reconstruction showed less clear chain-like arrangement (dashed arrow, Fig. 2f), likely due to the limited spatial resolution and the destructive sample preparation in APT. A proximity histogram concentration profile with a direction normal to the Si/Ni interface revealed a gold-enriched region (thickness, ~2 nm) localized at the Si sidewall surface (Fig. 2g), with an Au concentration as high as ~1120 atomic ppm. These results highlight the possibility of atom array patterning during a VLS process (Fig. 1).

### Ab initio molecular dynamics simulations

**Fig. 3:**
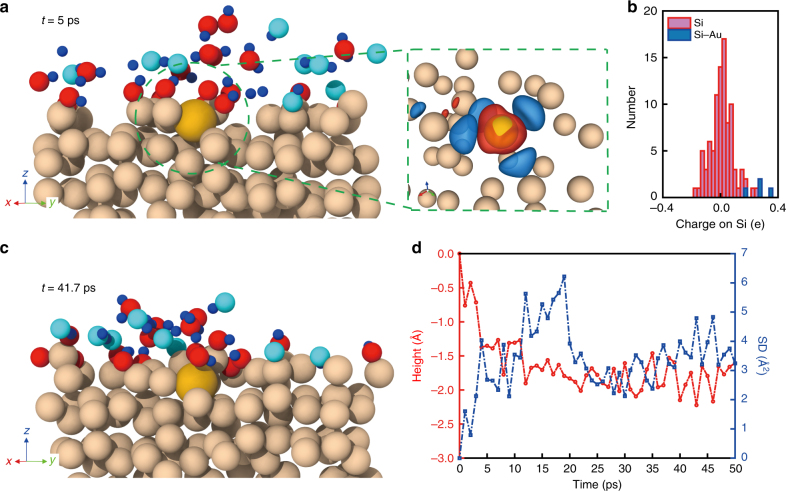
Ab initio molecular dynamics simulation of the catalytic effect of atomic Au. **a** A representative AIMD snapshot (left) and the corresponding charge transfer between Au and Si atoms (right) at *t* = 5 ps. Atoms are represented as almond (Si), yellow (Au), red (O), cyan (F), and blue (H) spheres. Isosurfaces represent volumes where electron density decreases (blue) and increases (red) due to the influence of the Au atom. **b** A histogram showing the distribution of charges on Si atoms bonded to Au (blue) and faraway bulk Si (red) atoms. Si atoms bonded to Au have the highest positive charges. **c** An AIMD snapshot sampled at a later time point, i.e., *t* = 41.7 ps. In both sampled configurations, the first nearest-neighbor Si atoms lose electrons to the more electronegative Au atom. This increase in electropositivity of adjacent Si atoms translates to higher reactivity in the presence of electron-rich species such as OH and HF. **d** Relative height and square displacement (SD) of the Au atom over the course of the simulation. Within the first 10 ps, the surface Au atom moved to the subsurface. An enhanced in-plane mobility was observed in the subsequent 15 ps

Parallel and ordered atom manipulation implies new opportunities in chemistry and applications for atomic metal-based interfaces. To explore this, we performed long-time ab initio molecular dynamics (AIMD) simulations of a model Si(111) surface with and without an isolated surface Au atom. Specifically, we wanted to assess the etching propensity of Si in an environment of hydrofluoric acid (HF), hydrogen peroxide (H_2_O_2_), and water (H_2_O) (see details in “Methods” section). This chemical process was chosen because it was relevant to the metal-assisted chemical etching (MACE) for porous Si^29–32^. These AIMD simulations were used to observe the dynamics of reactant molecules, as well as the structural evolution of Si in the vicinity of the Au atom (Fig. 3 and Supplementary Movies 1 and 2). In general, the Si(111) surface without an Au atom maintained structural integrity (Supplementary Fig. 4b), whereas the presence of a single Au atom showed pronounced disorder in the vicinity of the Au atom within the limited timescales accessible to AIMD (~50 ps) (Supplementary Fig. 4a).

To understand the role played by a single Au atom in driving the Si etching, we sampled several different configurations (Fig. 3a, c) from the long-time scale AIMD run and analyzed the evolving electronic structure of the Si substrate (in the vicinal regions, as well as those beyond the influence of Au atoms). Au being more electronegative than Si, it draws electrons from the Si atoms with which it bonds, thereby rendering the Si atoms slightly electron-deficient (Fig. 3a). A corresponding increase in the charge of Si bonded to Au was also seen in the distribution of Bader charges of Si atoms (Fig. 3b and “Methods” section), where Si bonded to Au (bars in blue) had the highest positive charges in the system. In two representative configurations (Fig. 3a, c), the Au atom received a charge of ~0.97 e, with the relative amounts of charge depletion on the neighboring Si atoms varying with time depending on the instantaneous structure. These positively charged Si atoms serve as active etching sites for the chemical species in solution, preferentially attracting electron-rich oxidizing agents such as hydrogen fluoride (HF) and hydroxyl group (OH). Such charge transfer does not occur in a Si(111) substrate without Au (Supplementary Fig. 4c). We note that the ease of etching Si in the presence of Au is also energetically favorable, given that the Au–Si bond dissociation energy is 15 kJ mol^−1^ lower compared to that for Si–Si^33^. Square displacement (SD) of the Au atom (Fig. 3d) suggested its significant mobility with an average diffusivity of ~5 × 10^−10^ m^2^ s^−1^, which was significantly higher than that for passive Au diffusion in bulk Si (<~10^−20^ m^2^ s^−1^)^34^. Additionally, height analysis of the Au atom (Fig. 3d) revealed its transport into the subsurface (Fig. 3c and Supplementary Fig. 4a) layers. This would enable continuation of the etching process after the removal of the surface Si atoms (beyond the timescales accessible to AIMD).

### Si nanowires with porous grooves

**Fig. 4:**
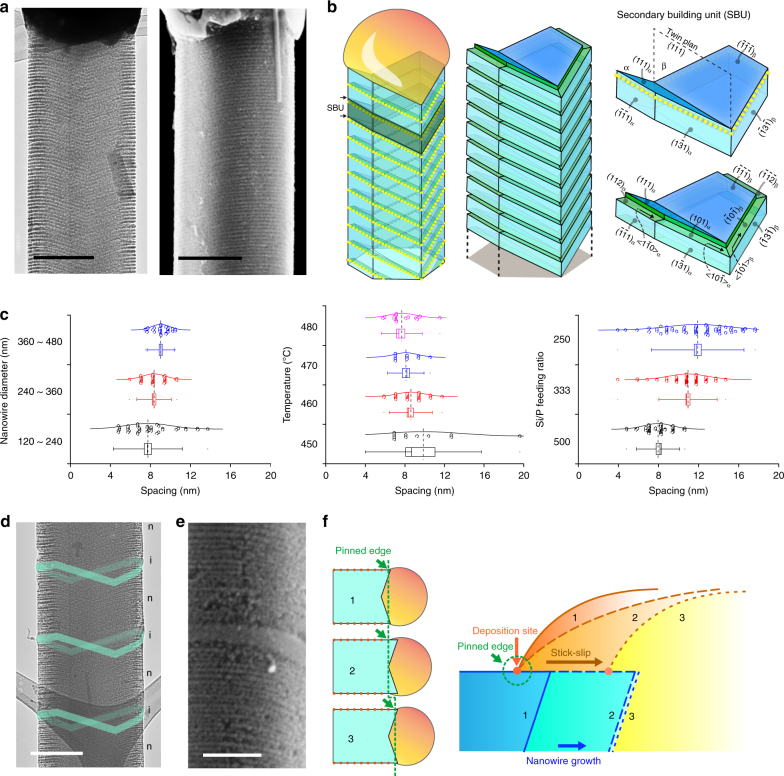
Atomic Au-catalyzed etching on Si nanowires. **a** TEM and SEM images showing ordered and 3D grooves over the entire *n*-type nanowire surfaces. Scale bars, 200 nm. **b** Schematics of the structure model of gold line patterns and the corresponding etched grooves on Si nanowire sidewall facets. Secondary building units before and after etching are highlighted. A twin plane is usually observed for <112> grown Si nanowires. **c** Statistical analyses of the groove spacing indicate the effects of the nanowire diameter (*n* = 36 (blue), 41 (red), and 43 (black)), growth temperature (*n* = 21 (magenta), 11 (blue), 32 (red), and 11(black)), and Si/P feeding ratio (*n* = 75 (blue), 92 (red), and 57 (black)) on the groove formation. Boxes, error bars, and dots represent standard errors, standard deviations, and maximum and minimal values, respectively. Dashed and solid lines represent mean and median values, respectively. **d**, **e** TEM and SEM images of etched dopant-modulated nanowires. Only the *n*-type segments yielded the grooves upon etching while the intrinsic segments (cyan ribbons in **d**, and the middle segment in **e**) remained smooth. Scale bars, 200 nm (**d**), 100 nm (**e**). **f** Schematic diagrams illustrating the mechanism for atomic Au line pattern formation. A model is proposed, combining a stick-slip motion and a Au deposition process. See Supplementary Discussion (analysis of atomic gold deposition) for details

The results from AIMD simulations suggest an atom-catalyzed etching to yield porous Si surfaces, an atomic version of classical MACE. We verified this process in wafer-scale and atomic Au-decorated Si nanowires for sub-10 nm^27^ and atom array-based catalytic lithography (i.e., atom deposition and catalytic etching) (Fig. 4). Transmission electron microscope (TEM) and scanning electron microscope (SEM) images recorded from etched *n*-type Si nanowires revealed massive, ordered, 3D, and porous grooves over all of the nanowire surfaces (Fig. 4a and Supplementary Fig. 5), reminiscent of ordered mesoporous materials^35, 36^. We observed similar etching behavior for nanowires that were stored in a desiccator for at least 6 months (Supplementary Fig. 6). Additionally, given that only atomic-scale Au was deposited, we did not observe obvious tapering of nanowires in our synthesis (Supplementary Fig. 7b). Upon etching, the yield of original atomic Au lines over *n*-type Si nanowires could be easily determined; they are ca. ~90% and ~30% for <112> and <111> growth orientations (growth condition at ~470 ^o^C, with a Si to P feeding ratio of ~500, and a diameter range of 120–480 nm) (Supplementary Fig. 7). Given that <112> orientation takes ~80% of the total nanowire population, the overall yield of Si nanowires that contained ordered atomic Au lines is ~78%. Due to their high growth percentage and etching yield, <112> nanowires will be the focus of our subsequent discussion.

TEM images and selected area electron diffraction (SAED) patterns recorded at different zone axes of <112> nanowires define a secondary building unit (SBU) and highlighted several key structural features (Fig. 4b and Supplementary Fig. 8a). First, we observed that all the original atomic Au lines ran along <110> directions over the sidewalls (yellow-dotted lines, Fig. 4b). Second, the etched porous grooves were placed on {110} facets if etching was initiated from the {113} sidewall, while the grooves sat on {112} facets if starting from the {111} sidewall (Fig. 4b). Third, the atomic Au lines and the corresponding grooves from {111} sidewalls were perpendicular to the nanowire growth axis, while those from {113} sidewalls were not (Fig. 4b).

Statistical imaging analysis with users’ script (Supplementary Fig. 9a, b and Supplementary Methods) indicated that larger nanowire diameter (e.g., > 120 nm), moderate temperature (e.g., 460–480 ^o^C), and a high phosphine concentration (e.g., Si/P feeding ratio < 1000) enabled high yield line and groove formation (Fig. 4c). While nanowire diameter and growth temperature (Fig. 4c) did not significantly affect the average groove spacing, the distribution became narrower under certain conditions (i.e., 360–480-nm diameter and 470 ^o^C). Phosphine concentration (Supplementary Fig. 9c), however, had a significant effect, with a Si/P feeding ratio > 2000 yielding no grooves or atomic Au lines, and an increased groove spacing and wider distribution when the Si/P feeding ratio varied from 500 to 250 (Fig. 4c).

Different from *n*-type Si nanowires, intrinsic and *p*-type nanowires do not yield ordered grooves upon etching (Fig. 4d, e). When *n*-type/intrinsic dopant modulation was employed, we observed a < 10-nm sharp transition (Fig. 4e) from parallel grooves (i.e., *n*-type Si) to a smooth surface (i.e., intrinsic Si). These facts suggest that the alloy droplet dynamics are highly sensitive to their immediate chemical environment near the triple-phase boundary (TPB), which can be switched reversibly and quickly between multiple states.

### Mechanistic understanding

The easier imaging and enhanced clarity of etched grooves enable a mechanistic understanding of the underlying atomic Au pattern formation (Fig. 4f). Specifically, we sought to address the following questions. First, why do the <112>-oriented Si nanowires show a much higher patterning yield than <111>-oriented ones, i.e., ~90% vs. ~30%? Second, why do dopants exert a significant impact? And specifically, why does only phosphine yield the observed line patterns? Finally, what is the built-in “switching” mechanism to regulate the Au deposition during an *n*-type Si nanowire growth?

Regarding the orientation-dependent line pattern yield, we noted two structural differences between <112>- and <111>-oriented nanowires. In <112>-oriented nanowires, there are two exposed {111} facets and four exposed {113} that are known to promote wetting or accumulation of Au species^11, 14, 37–39^. Moreover, essentially all <112>-oriented Si nanowires display at least one {111} twin plane that runs parallel to the nanowire axis^11^; such a planar defect is known to accumulate gold species^40^. <111>-oriented nanowires, however, do not have {111} sidewall facets (unless it has a sawtooth geometry^22, 39^ which was not the case under our growth condition) and only occasionally contain inclined {111} faults that occupy a small portion of nanowires^37, 38^. These two factors together suggest that the sidewalls of <112>-oriented nanowires (that contain {111} and {113} facets and exposed twin plane edges) are more sticky to Au/Si alloy droplets and promote the droplet contact to the sidewall^41^ and subsequent atomic Au line patterning. <111>-oriented nanowires do not have significant sticky features, giving lower yield or incoherent line patterns, or line patterns that are accumulated primarily around the inclined {111} faults (Supplementary Fig. 10).

Next, we only observed ordered Au lines in phosphorus-doped (i.e., *n*-type) Si nanowires, but not in boron-doped (i.e., *p*-type) or intrinsic Si nanowires. This can be understood by considering two criteria that must be satisfied simultaneously: (I) the as-deposited Au should be patterned, and (II) the as-deposited Au should be immobilized/stabilized so that the patterns would not randomize during the later stage of synthesis.

Without in situ imaging tools, we cannot rule out the possibility that the as-deposited atomic Au in intrinsic, boron-doped, and phosphorus-doped Si nanowires would all be ordered, i.e., criteria I may be satisfied for intrinsic and doped nanowires. However, for criteria II, different surface chemistry can have drastic effects on the stability of as-deposited Au. The boron (i.e., a *p*-type dopant) precursor was shown to promote Au diffusion along Si nanowire sidewalls^11^, which produced silicon spicules with graded gold coverage. This fact suggests that boron cannot immobilize atomic Au. For intrinsic Si nanowires, the sidewall was passivated with atomic hydrogen, which typically inhibited Au deposition. Even if Au atoms were deposited over the sidewall during the synthesis, they tended to quickly de-wet and form nanoparticle aggregates^11^. Finally, phosphorus, as decomposed from phosphine (PH_3_, an *n*-type dopant precursor), is known to interact strongly with both Si and Au^16, 33, 42^ and preferentially accumulate around the nanowire surfaces in *n*-type Si nanowires^43^. Indeed, previous results on *n*-type silicon spicules indicate minimum gold-based gradient along Si nanowire sidewalls^11^, confirming the immobilization role of phosphorus over Au. Overall, only phosphorus-doped Si nanowires can satisfy criteria II, i.e., stabilizing the as-deposited ordered atomic Au lines.

Phosphorus exists in multiple phases of the nanowire system. The key question is, “Which part of phosphorus is critical?” The sharp pattern transition between phosphorus-doped and undoped segments (Fig. 4e) suggests that the dopant reservoir effect^44^, which usually covers a characteristic length of approximately the nanowire diameter, would not impact the line patterning. Therefore, phosphorus inside the liquid alloy droplet (to be used for doping) and the solid Si nanowire (already used for bulk or subsurface doping) is not critical for the observed Au patterns. Instead, only phosphorus in the gas phase and over the silicon surfaces regulates the pattern formation.

Finally, regarding the built-in switching mechanism for atom printing, it requires that the VLS growth system allows both the alloy-based nozzle positioning and atomic Au-based ink delivery. As shown below, we propose that a stick-slip motion and a chemical potential variation enable the positioning and the delivery (Fig. 4f and Supplementary Fig. 11), respectively.

As stated earlier, phosphorus binds strongly with Au and Si nanowires^16, 33, 42^. Such a robust interaction would cause pinning of the alloy droplet (Supplementary Discussion) at the Si sidewall with a pinning potential barrier (*U*), as well as the immobilization of as-deposited atomic Au so as to maintain the patterns. During nanowire growth (Fig. 4f and Supplementary Fig. 11), the alloy droplet contact angle (*θ*) decreases initially because the TPB is pinned at the sidewall (i.e., “Stick”). When the *θ* reaches its minimum (*θ*_min_), the potential barrier *U* can subsequently be overcome by the gain in Gibbs free energy (Δ*G*) upon snapping to the next equilibrium/quasi-equilibrium position with a contact angle *θ*_0_ (i.e., “Slip”)^45^; see details in Supplementary Discussion. This droplet motion can position the droplet edge (the “nozzle”).

Because Si chemical potential difference near the TPB is proportional to contact angle *θ* (Supplementary Discussion)^21, 46^, a reduction of *θ* upon nanowire elongation caused a local chemical potential variation, and correspondingly, a transfer of Au atoms (i.e., “ink”) from the liquid alloy to the solid Si surfaces near the TPB. This angle-dependent atom deposition is reminiscent of the coffee-ring effect. However, unlike the traditional coffee-ring process where evaporation of the droplet causes *θ* reduction and capillary flow, the driving force for the *θ* dynamics and atomic Au deposition in our case was the elongation of the Si nanowire and the corresponding chemical potential changes at TPB. Iterations of this *θ* variation then yielded the observed parallel atomic Au line patterns on Si nanowire sidewalls.

### Ex situ studies of complex crystal growths

**Fig. 5:**
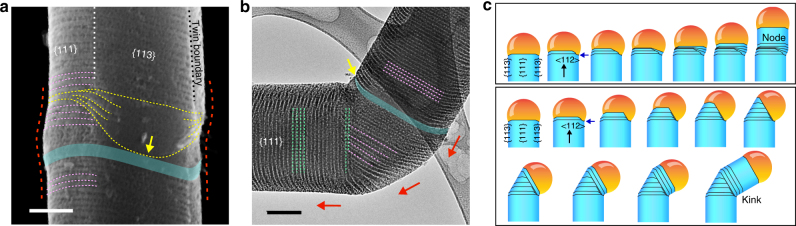
Atomic gold patterns enable the study of the growth dynamics of existing structures. **a** A SEM image of a nanowire with a node. Yellow and pink dashed lines mark the evolution of the alloy droplet during node formation. The yellow arrow marks the pinning edge of the droplet on an intrinsic segment (cyan ribbon). Red dashed lines highlight the node evolution. Scale bar, 100 nm. **b** A TEM image of a kinked nanowire. Red arrows indicate the switches of the nanowire growth orientations. The yellow arrow marks the pinning edge of the droplet on the sidewall at an intrinsic segment (cyan ribbon). Pink and green dashed lines highlight the grooves in the original and the new arms, respectively. Scale bar, 100 nm. **c** Schematics of the growth dynamics. A node is formed when the droplet edge first gets pinned heavily on one facet (blue arrow) while the rest of the droplet continues to evolve. Subsequent unpinning recovers the original growth behavior, leaving a node behind. In the formation of a kinked unit, the pinned droplet edge remains attached to the Si sidewall (blue arrow), while the growth orientation switches between two <112> directions by shrinking/enlarging the Au/Si alloy droplet/Si nanowire interfaces that yield the original/new arm orientations

**Fig. 6:**
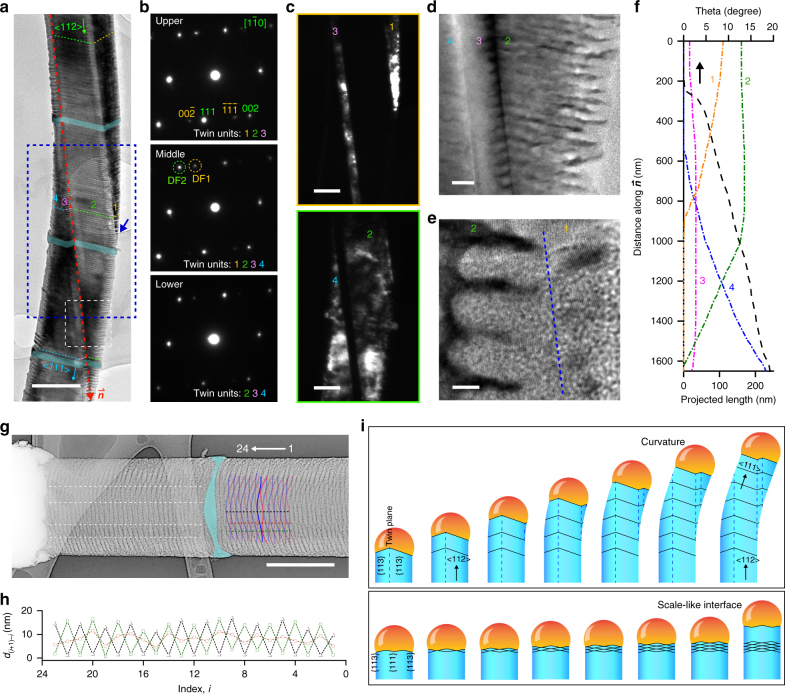
Atomic gold patterns enable the discovery of new crystal growth behaviors. **a** A BF TEM image of the nanowire with multiple twin units labeled by numbers. The growth orientation is shifted gradually from <112> to <111>. Cyan ribbons highlight the intrinsic segments. Scale bar, 200 nm. **b** SAED patterns taken at upper, middle, and lower portions of the nanowire in **a**. **c** DF TEM images formed by selecting diffraction spots marked by yellow (DF1) and green (DF2) dashed circles in **b**. Scale bars, 100 nm. **d**, **e** HRTEM images from different regions in **a**, marked by a white dashed box (**d**) and a blue arrow (**e**). Scale bars, 20 nm (**d**), 5 nm (**e**). **f** Growth behavior analysis shows a smooth transition of the nanowire orientation from a <112> (marked by $\mathop{{\boldsymbol{n}}}\limits^{\rightharpoonup}$ in **a**) to a <111> direction. Projected lengths of individual twin units and the nanowire angular orientation are plotted along $\mathop{{\boldsymbol{n}}}\limits^{\rightharpoonup}$. **g** A TEM image (with color inversion) of a nanowire with a scale-like TPB. Scale bar, 200 nm. **h** The growth behavior is analyzed by tracking spacing *d*_(*i*+1)–*i*_ between adjacent wavy lines (blue and pink lines) at three different locations (black, brown, and green dashed lines in **g**). The white arrow in **g** indicates the direction of analysis. **i** Schematics of the growth dynamics. A curved nanowire can be formed when it gradually switches the growth orientation from <112> to <111> (black arrows), without involving a kink unit. During the transition, the droplet crosses multiple twin boundaries, marked by black dashed lines. A scale-like alloy/Si interface is formed when the TPB oscillates between two wavy line shapes. The cyan ribbon in **a** and **g** marks an intrinsic segment

Since the grooves faithfully delineated the 3D TPB geometry, tracing these features can imply complex material growth dynamics (Figs. 5 and 6), which are typically studied by in situ techniques^20, 21, 47^ in a constrained environment. For these studies, we intentionally introduced narrow intrinsic segments (cyan bands, Figs. 5a, b and 6a, g), as their smooth surfaces upon etching can either mark the locations of growth perturbations (Fig. 5a, b) or help evaluate the stability of structural evolution (Fig. 6a, g).

We first studied two complex building blocks, nodes and kinks^48^ (Fig. 5). We found that during node growth (Fig. 5a, c), the droplet edge first pinned heavily on one facet upon dopant switching (or growth perturbation), while the rest of the droplet continued to evolve (pink dashed lines). Subsequent unpinning (yellow dashed lines, Fig. 5a) recovered the original growth behavior, leaving a node behind (red dashed line in Fig. 5a, c). However, for a kinked unit (Fig. 5b, c), the pinned droplet edge remained attached to the Si sidewall (yellow arrow), while the growth orientation switched between two <112> (red arrows) by shrinking/enlarging the droplet/Si interfaces that are parallel to the original/new ones (i.e., highlighted in pink and green dashed lines, respectively). These observations are consistent with previous models^37, 48, 49^.

With this atom-enabled etching, we also discovered two new growth behaviors (Fig. 6). First, we found that a Si/Au alloy droplet crossing multiple twinning units (labeled as 1, 2, 3, and 4) can gradually switch the growth orientation from <112> to <111> (Fig. 6a–f), without involving a kinked unit. SAED from different portions (Fig. 6b) and dark-field TEM images from selected SAED spots (Fig. 6c) showed the existence and size evolution for each unit. Zoom-in TEM images (Fig. 6d, e) revealed sharp twin boundaries (Fig. 6d) and the hierarchical nanoscale and atomic-scale ordering (Fig. 6e). The TPB showed zigzag shapes (Fig. 6d), and the projected lengths for each TPB segment and the nanowire angular orientation changed smoothly during the <112> to <111> transition (Fig. 6f). This observation highlights an unusual curvature formation mechanism, i.e., through the size evolution of multitwinned units in crystalline materials (Fig. 6i, upper panel). Second, the scale-like TPB was observed when the phosphine concentration was high (100 < Si/P feeding ratio <300), with a typical yield of <15%, together with a majority of parallel TPB patterns (Figs. 2, 4, and 5). The TPB oscillated between two wavy line shapes (red and blue lines in Fig. 6g, lower panel in Fig. 6i), and the spacing between adjacent TPB lines (*d*_(*i*+1)*–i*_) fluctuated (versus being mostly constant for parallel TPB patterns) and was location-dependent (Fig. 6h, recorded along black, brown, and green dashed lines in Fig. 6g). Finally, in both curvature-forming TPB (Fig. 6a) and scale-like TPB (Fig. 6g), the insertion of intrinsic segments (cyan bands) did not terminate the material growth behaviors.

## Discussion

In this work, we demonstrated an approach for parallel patterning/printing of individual atoms over smooth substrates. We revealed an atomic version of MACE, where single Au atoms can catalyze the etching of Si to create < 5-nm features. We also discovered an alloy droplet instability during the classical VLS growth which would promote a stick-slip motion along the nanowire sidewalls. Finally, using atom patterning and atom-catalyzed etching, we revealed several complex crystal growth dynamics that are hard to probe in the past.

In particular, the previous alloy droplet instabilities in VLS systems are all related to a configuration where the liquid is supported by the nanowire end facets^20–24^. In this work, the new droplet instability is relevant to droplet wetting over the nanowire sidewall^41^. This wetting configuration has received very little attention in the past, although theoretical analysis has predicted its existence^41^.

There are several important experimental factors that contribute to a series of observations in this work. First, the use of STEM imaging from thin nanowire sections (by microtoming) is important. Even under STEM, the atomic Au lines are only visible with a careful tilting of the sample. Traditional TEM cannot reveal such atomic Au lines unless chemical etching is used to amplify their original locations (Figs. 4–6). Therefore, the phenomena revealed in this work may have existed for a very long time, but were overlooked due to nonideal imaging techniques used before. Second, we only observed Au patterns in large-diameter Si nanowires (>120 nm), which is beyond the most studied diameter range of 5 ~100 nm. This fact is consistent with the prior studies that larger-diameter Si nanowires tend to accumulate more Au^14, 15^. Third, both the nanowire orientation and doping type are critical for the high yield deposition of Au lines, which are discussed in detail in the “Results” section. Most prior studies on Si nanowires have been focused on <111> orientation, instead of the <112> in the current work. Finally, the atomic-scale MACE revealed in this work also facilitates the discovery and understanding of this new droplet instability. This atomic-scale catalytic etching highlights the location of the original Au lines.

With regard to potential applications, the atom-based self-labeling may be used to reveal the growth mechanisms for many other complex nanowire structures, such as epitaxial lateral nanowires^50^ and island-chain nanowires^51^. This labeling may be broadly applied to chemistry and materials science with a role analogous to the use of fluorescent molecules in probing biological dynamics. Additionally, the parallel atom deposition and etching over nanowire sidewalls may be extended to atom-based catalytic lithography over planar substrates.

## Methods

### Synthesis of *n*-type silicon (Si) nanowires

Silicon nanowires were synthesized using a gold (Au) nanocluster-catalyzed chemical vapor deposition (CVD) method. The citrate-stabilized Au colloidal nanoparticles (Ted Pella, USA, 200 nm) were deposited onto Si (100) substrates (Nova Electronic Materials, USA, *n*-type, 0.001–0.005 Ω cm) as catalysts. Prior to the catalyst deposition, native oxide layers on the Si substrates were removed with hydrofluoric acid (HF) (Sigma-Aldrich, USA) to yield hydrogen-terminated surfaces. The growth of Si nanowires was effectuated at 450–490 ^o^C using silane (SiH_4_) as the silicon source, phosphine (PH_3_, 1000 ppm in H_2_) as the *n*-type dopant, and hydrogen (H_2_) as the carrier gas. In a typical synthesis of *n*-type Si nanowires with a Si/P feeding ratio of 500:1, the flow rates of SiH_4_, PH_3_, and H_2_ were 2, 4, and 60 standard cubic centimeters per minute (sccm), respectively. The growth chamber pressure was maintained at 40 Torr throughout the synthesis. The Si/P feeding ratio was varied by changing the flow rate of PH_3_ between 2 sccm and 8 sccm while fixing the SiH_4_ flow rate as 2 sccm to reach Si/P feeding ratios from 1000:1 to 250:1.

### Synthesis of doping and pressure-modulated Si nanowires

In a typical growth, an *n*-type base segment was grown at 470 ^o^C with a Si/P feeding ratio of 500:1 by setting the flow rates of SiH_4_, PH_3_, and H_2_ as 2, 4, and 60 sccm, respectively. The base segment was grown under a constant pressure of 40 Torr for 1 min. Later on, periodic switches between 7 s of evacuation and 23 s of pressure ramping were performed to modulate the growth chamber pressure during the nanowire growth. During 23 s of pressure-ramping periods, the PH_3_ flow was turned off in the first 3 s to modulate the doping level of the nanowires. The pressure-switching cycles were iterated for 10 min after the base segment growth to yield doping and pressure-modulated Si nanowires.

### Ab initio molecular dynamics simulations

We performed ab initio molecular dynamics (AIMD) simulations within the generalized gradient approximation (GGA) using the projector-augmented wave formalism, as implemented in the Vienna Ab initio Simulation Package (VASP)^52^. We used the empirical method of Grimme^53^ to account for vdW interactions. The exchange correlation was described by the Perdew–Burke–Ernzerhof (PBE) functional^54^, with the pseudopotentials supplied by VASP. The plane wave energy cutoff was set at 400 eV. The Brillouin zone was sampled at the Γ-point only. The computational supercell (96 atoms) consisted of diamond cubic Si oriented such that X (1–10), Y (11–2), and Z (111). An Au atom was placed on an Si site in the initial structure. Periodic boundary conditions were employed along all directions. A vacuum of 20 Å was introduced in the Z direction to model the (111) surface. HF and H_2_O_2_ (10 molecules of each) were placed randomly on the (111) surface of Si. Subsequently, we performed AIMD simulations in the canonical (NVT) ensemble at 600 K via the Nosé–Hoover thermostat^55^ as implemented in VASP, using a time step of 0.5 fs to update the atom positions.

### Etching of the Si nanowires

The parallel atomic gold-based line patterns on Si nanowires were revealed using an etching system consisting of HF and hydrogen peroxide (H_2_O_2_). In a typical etching experiment, the as-grown Si nanowire substrates were dipped into a 10.85 wt% H_2_O_2_ (Fisher Scientific, USA) and 10.37 wt% HF aqueous mixture solution at 10 °C for 8–45 s. The etched samples were rinsed with deionized (DI) water, isopropyl alcohol (IPA), and blown dry by nitrogen (N_2_).

### Microscopy analysis

Unetched Si nanowires were sectioned using an epoxy resin-based ultramicrotome technique for the study of Au distribution on nanowire surfaces by STEM. Nanowires embedded with epoxy resin precursors (low-viscosity Spurr, Ted Pella, USA) were first solidified at 60 °C for 24 h. Resin sections of ~70 nm were cut by an ultramicrotome (Ultracut E, Reichert-Jung, USA) and collected by lacey carbon copper grids (Ted Pella, USA, Lacey Carbon, 200 mesh). The high-angle annular dark-field (HAADF) STEM images of sectioned Si nanowires were taken on an aberration-corrected STEM operated at 200 keV (JEM-ARM200CF, JEOL, Japan). The electron-dispersive X-ray (EDX) spectra were obtained using an Oxford X-Max^N^ 100TLE windowless SDD X-ray detector (Oxford Instruments, UK) on the same JEOL STEM while imaging. For electron microscopy of etched Si nanowires, they were gently sonicated in IPA and dispersed onto silicon substrates (Nova Electronic Materials, USA, *n*-type, 0.001–0.005 Ω cm) or lacey carbon copper grids. A Carl Zeiss SEM (Merlin FE-SEM, Carl Zeiss, Germany) and an FEI TEM (Tecnai F30, FEI, USA) were used to characterize the morphology of etched Si nanowires. Crystallography of the Si nanowires was studied by analyzing bright-field (BF) and dark-field (DF) TEM images and selected area electron diffraction (SAED) patterns taken by a JEOL TEM operated at 300 keV (JEM-3010, JEOL, Japan). DF TEM images in Fig. 6c were formed by tilting the electron beam to position the corresponding diffracted beams in Fig. 6b, marked by DF1 and DF2, into the objective aperture to highlight individual twin units. A Hitachi HD-2300A STEM (Hitachi, Japan) was used to study the etched morphology in three dimensions (3D) by collecting tilting STEM series. Au nanoparticles of 10 nm were applied onto Si nanowires as fiducial markers. Etched Si nanowires on Si substrates were first picked up and transferred with a micromanipulator and attached to copper (Cu) tips with electron-beam-induced platinum (Pt)/carbon (C) deposition using a focused ion beam (FIB) system (FEI, USA, Helios Nanolab 600 DualBeam FIB/SEM). The HAADF-STEM images of the samples were collected at tilt intervals of 2° from 0° up to 210°. For atomic force microscopy (AFM), unetched Si nanowires were transferred to Si substrates (Nova Electronic Materials, USA, *n*-type, 0.001–0.005 Ω cm) and imaged using an Asylum Cypher AFM (Asylum Research, USA).

### Atom-probe tomography (APT)

Unetched *n*-type Si nanowire surfaces were first protected with a 50-nm Ni-capping layer deposited by an electron beam evaporator (AJA International, USA). The nanowires were then transferred horizontally onto silicon microposts using a micromanipulator, mounted with Pt/C deposition, and milled into needle-like microtip specimens using a FEI Helios Nanolab 600 FIB (FEI, USA) system. APT was performed using an ultraviolet (UV) laser-assisted local-electrode atom probe (Cameca, USA, LEAP 4000X Si). Surface atoms from the microtips were field evaporated with an applied voltage of 1–7-kV direct current (dc) assisted with a UV (wavelength *λ* = 355 nm) laser pulsing at 250 kHz with a pulse energy of 20 pJ. The mass-to-charge (*m*/*z*) ratios of individual field-evaporated ions, in addition to their (*x*, *y*, *z*) coordinates in direct space, were recorded by a position-sensitive time-of-flight detector. During the APT analyses, samples were held at a 30-K base temperature at an ambient pressure of ~2 × 10^−11^ Torr. The 3D reconstructions of the data sets were performed utilizing Cameca’s IVAS 3.6 code. In a typical analysis, a region-of-interest (ROI) selection tool was used to isolate a region that included the nickel (Ni)-capping layer, the native surface silicon oxide layer (SiO_2_), and a layer of the Si nanowires. A 2D concentration map of Au was created by analyzing the number of Au atoms per unit volume and projecting the density map on the Si/SiO_2_/Ni interface plane. An 80 at% Si isoconcentration surface was created to delineate the region where the Si atomic concentration on one side was larger than 80 at%. A proximity histogram concentration profile^56^ was created by analyzing concentrations of individual atoms per unit length, beginning in the Ni layer and moving along the normal direction to the Si isoconcentration surface into the Si nanowire.

### X-ray photoelectron spectroscopy (XPS)

Four planar Si substrates with different surface treatments were used for XPS analysis. A planar Si substrate (Nova Electronic Materials, USA, *n*-type, 0.001–0.005 Ω cm) with only HF treating for native oxide removal was used as a control sample. The second sample was HF treated before dipping in an Au etchant for 4 min and a 10.85 wt% H_2_O_2_ solution for another 15 s at 10 °C. On the third sample, 200-nm Au nanoparticles (Ted Pella, USA) were deposited on the HF-treated Si surface. The Au-deposited Si substrate was annealed in a CVD system under vacuum at 700 °C for 5 min. Finally, the fourth sample underwent the same processing condition with the third one for Au diffusion on Si. It was then dipped in an Au etchant for 4 min before being transferred into a 10.85 wt% H_2_O_2_ solution for 15 s at 10 °C. A Kratos AXIS Nova system was used for XPS analysis (Kratos, UK) with a monochromatic Al Kα (*hv* = 1486.6 eV) source. The Al anode, for all measurements, was at 10 mA and 15 kV. The analysis spot of the X-ray beam was 300 × 700 µm^2^. Samples were calibrated via the Si 2p peaks located at 99.8 eV. The C 1 s peak at 285.5 eV was also used to support this calibration. Pt metal was added to the surface of a number of samples so that the Pt 4 f signal at 71.0 eV could verify the calibration. Surveys were collected at 160-eV pass energy and a step size of 1 eV. The high-resolution spectra for Si 2p were collected at 20-eV pass energy, a step size of 0.1 eV, 120-s sweeps, and with 3 and 20 sweeps, respectively.

### Data availability

The data sets analyzed during the current study are available from the corresponding author on reasonable request.

## Electronic supplementary material

Supplementary Information

Description of Additional Supplementary Information

Supplementary Movie 1

Supplementary Movie 2
