# Supplementary material for: Alloy-assisted deposition of three-dimensional arrays of atomic gold catalyst for crystal growth studies
Source: Nat Commun. 2017 Dec 8;8:2014. doi: 10.1038/s41467-017-02025-x (PMC5722855; doi:10.1038/s41467-017-02025-x)
Supplement: Supplementary file 1 — Supplementary Information [file 41467_2017_2025_MOESM1_ESM.pdf]

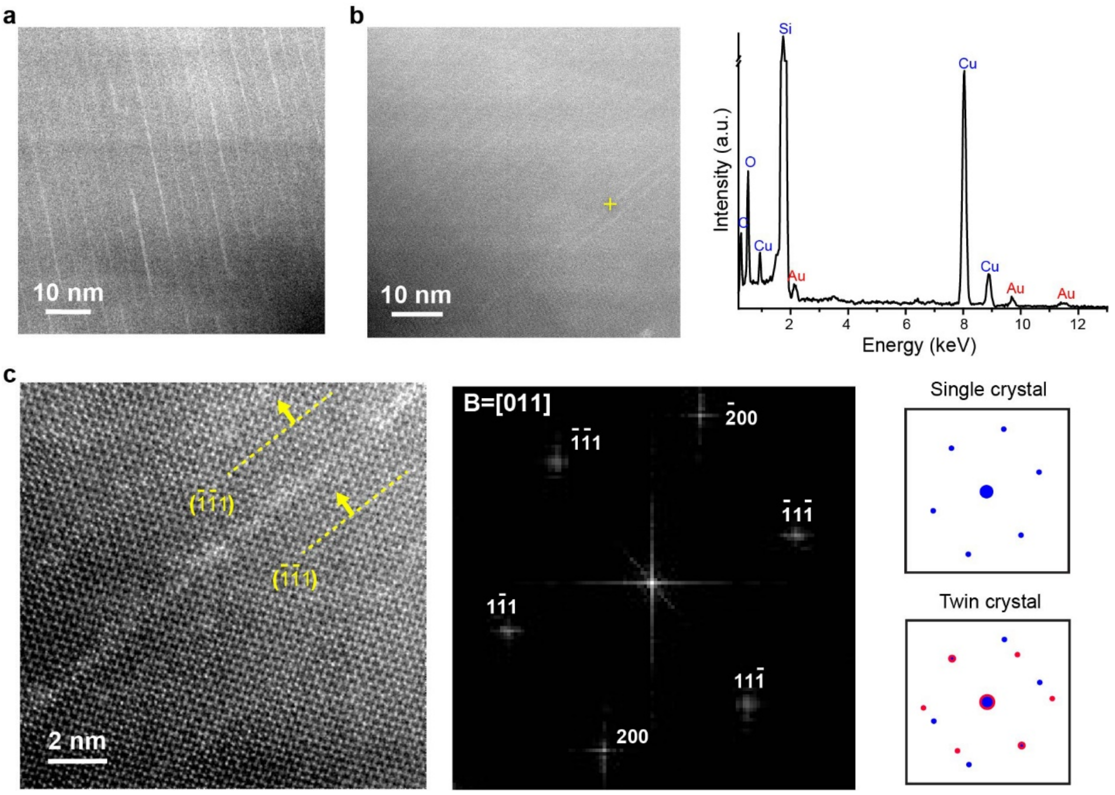

Supplementary Figure 1 | STEM and EDX studies of ordered line patterns on Si nanowire surfaces. a, Ordered line patterns recorded from a different Si nanowire sample. **b,** A STEM image and an EDX spectrum taken from the same Si nanowire used for **Fig. 2b**. Characteristic x-ray peaks of Au were identified. The yellow cross on the STEM image (left) marks the spot where the EDX spectrum was collected. **c,** A high-resolution HAADF-STEM image (left) and its corresponding fast Fourier transform (FFT) diffractogram (middle) show a single crystalline characteristic when viewed from [011] zone. The fact that Au lines are not aligned with any {111} twin boundaries excludes the possibility that the line patterns are formed by trapped Au atoms in a twinning superlattice structure. A simulated FFT from a twinning superlattice (lower right panel) should show two sets of diffraction spots (red and blue), which are not identified in our case (middle and upper right panels).

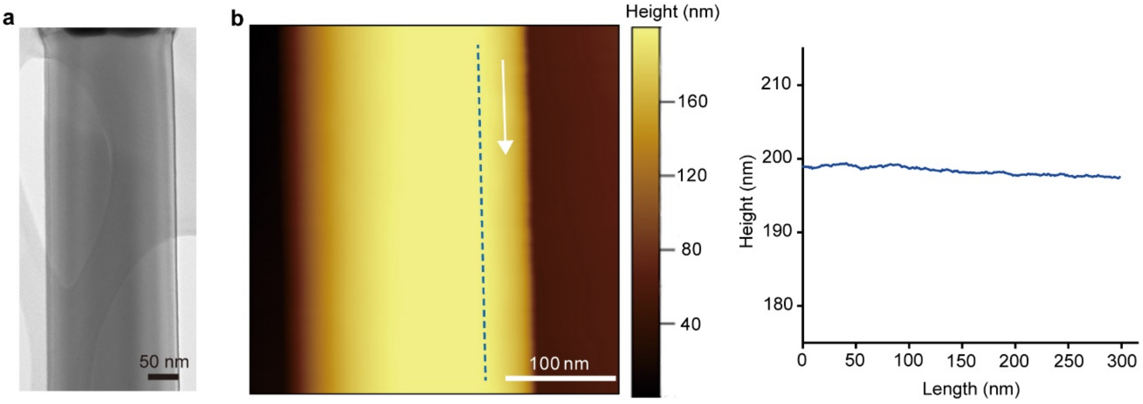

Supplementary Figure 2 | As-grown Si nanowires have smooth surfaces. TEM (**a**) and AFM (**b**) images show that the surfaces of *n*-type Si nanowires are smooth, without sawtooth sidewall faceting. A height profile taken along the nanowire axial direction (**b**, right) quantifies the observed surface smoothness.

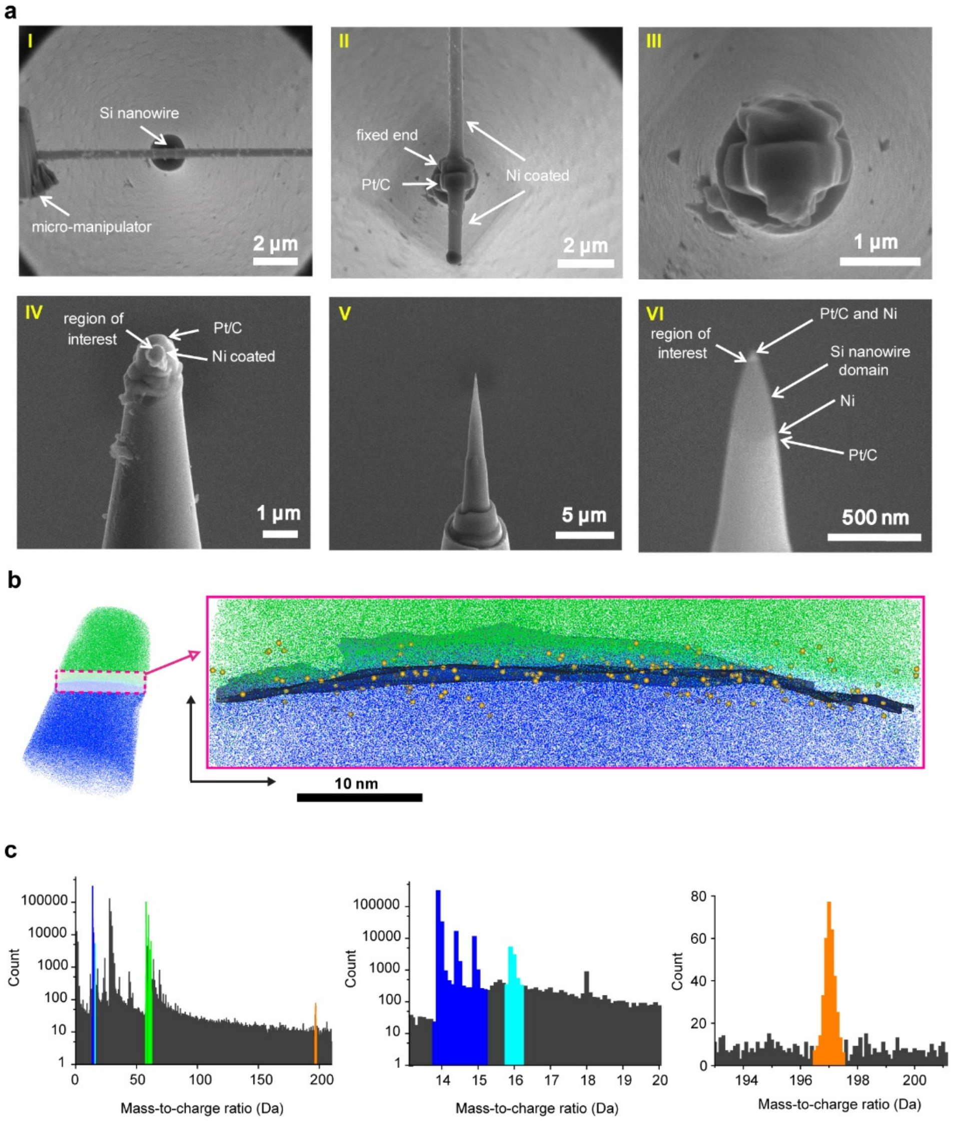

Supplementary Figure 3 | APT specimen fabrication process, data reconstruction and analysis. a, An unetched *n*-type Si nanowire with a Ni surface protection layer is placed horizontally onto a silicon micropost using a micromanipulator. After mounting with Pt/C deposition, the region of interest on the nanowire is milled into needle-like microtip specimens using a focused-ion beam instrument. **b,** An atom-by-atom 3-D reconstructed data set of the Si nanowire specimen (Si, blue dots, 4% shown) with the SiO_2 (O, cyan dots, 75%) native surface oxide and the Ni (Ni, green dots, 7.5%) capping layer (left). The pink dashed box mark the region for the enlarged cross-sectional view of the Si/SiO_2/Ni interfaces (right). An 80 at% Si isoconcentration surface (blue) delineates the Si/SiO_2 interface. 20% of total Si (blue dots), 100% of O (cyan dots), 75% of Ni (green dots), and 100% of Au (yellow spheres) atoms are displayed to highlight the enrichment of Au at the Si nanowire surface. **c,** Mass spectra of the interfacial region (**b**, right) shows a distinct peak for the ^{197}Au isotope, corroborating the EDX results that suggest the presence of Au atoms on the nanowire surface.

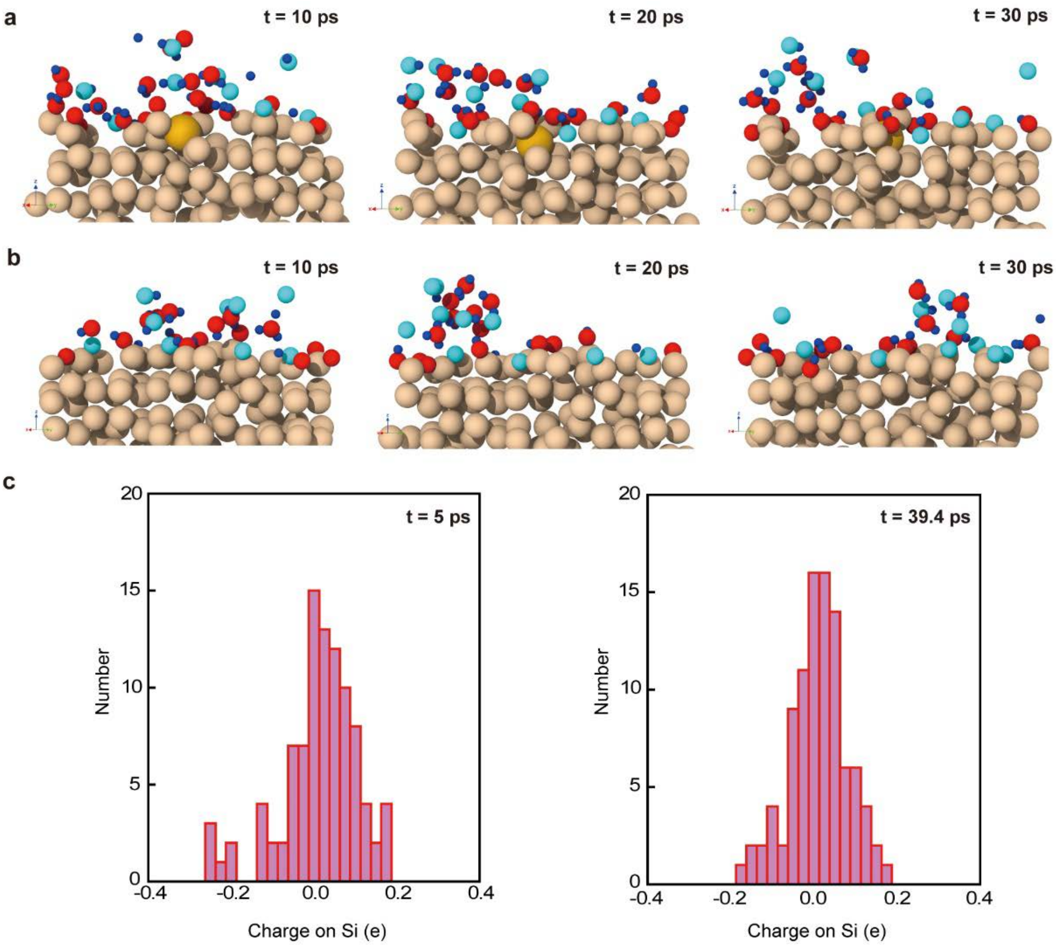

Supplementary Figure 4 | AIMD snapshots (10, 20 and 30 ps) in Si(111) with atomic gold and distribution of Si charges in pure Si(111) system. a. Single Au atom caused local disorder and showed significant mobility in Si. Atoms are represented as almond (Si), yellow (Au), red (O), cyan (F), and blue (H) spheres. **b**. The Si(111) surface without Au atom maintains its structural integrity. **c.** Charge histograms for Si atoms in the Si(111) substrate without Au atom, at 5 ps (left panel) and 39.4 ps (right panel), respectively. Compared with Si atoms bonded to Au, Si atoms with large positive charges are not observed during the course of the AIMD simulations.

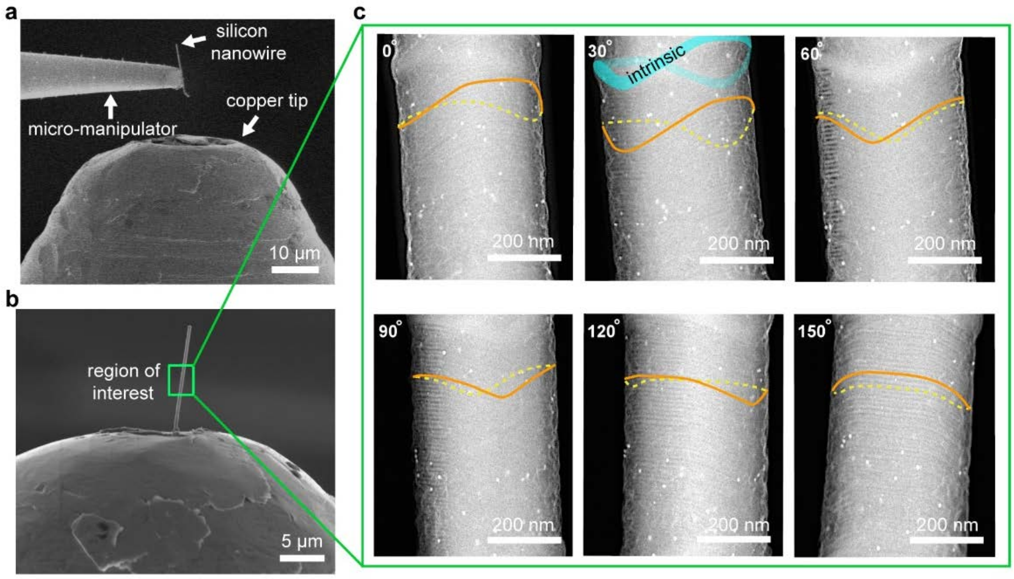

Supplementary Figure 5 | HAADF-STEM tilting series show the 3-D grooves on Si nanowire facets. a, An SEM image showing a Si nanowire picked up by a micromanipulator. **b,** The nanowire was mounted on a Cu tip. The green box marks the region of interest for the STEM tilting image series. **c,** HAADF-STEM images of the same nanowire imaged from six different perspectives. Relative tilting angles are marked in individual panels. Orange (top surface) and dashed yellow (bottom surface) lines mark the orientations of the etched grooves. The cyan ribbon highlights the shape of an intrinsic segment.

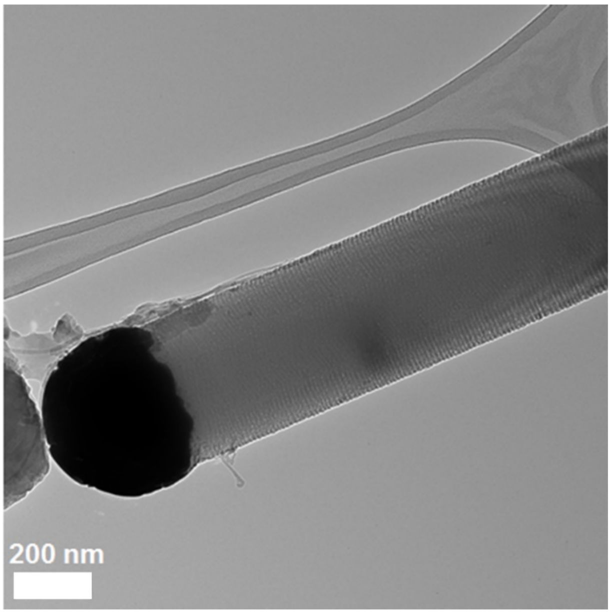

Supplementary Figure 6 | A TEM image of an etched nanowire which was synthesized about 6 months prior to etching and imaging. We stored the nanowires in desiccators. We got similar groove structures as those from freshly prepared samples.

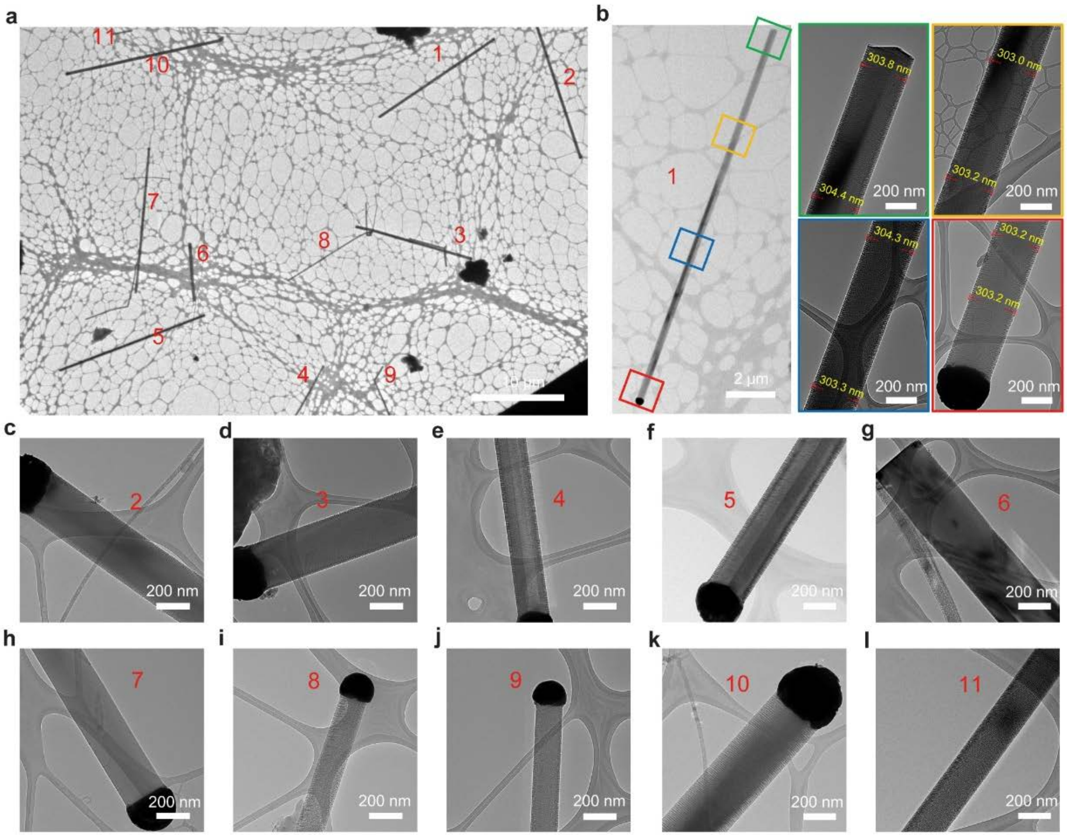

Supplementary Figure 7 | Si nanowires with ordered grooves display high yield and minimal tapering. a, A TEM overview of etched *n*-type Si nanowires. Numbers correspond to individual nanowires with zoom-in views. **b,** An etched Si nanowire with TEM images taken at multiple locations along its axial direction. Ordered grooves were observed on the entire nanowire with marginal differences. Minimal nanowire tapering was observed. **c-l,** TEM images of individual nanowires showing a high yield of ordered grooves. Among 11 nanowires, only #6 and #7 did not show ordered grooves.

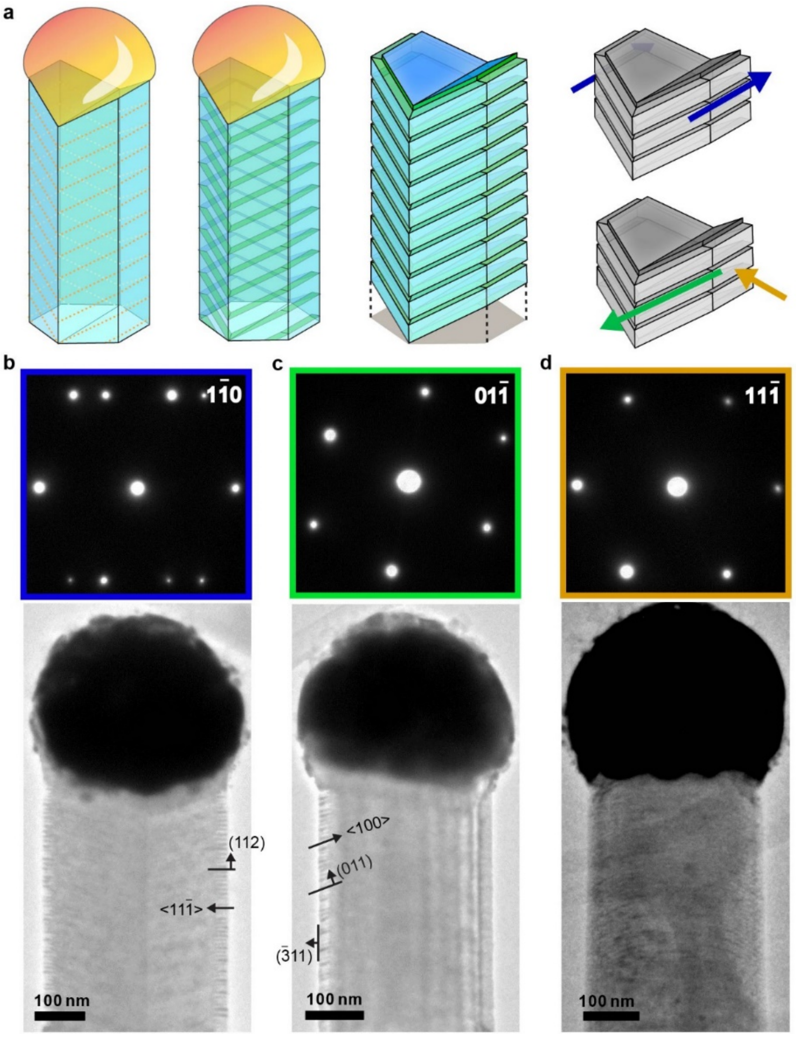

Supplementary Figure 8 | Structures of etched grooves on <112> grown Si nanowires. a, Ordered gold line patterns (first from left) and corresponding etched grooves (second and third from left) follow the contour shape of the interfaces between the Au/Si liquid alloy and the Si nanowire. All the gold lines and corresponding etched grooves run along the <110> directions. Three secondary building units are stacked (fourth from left) and different viewing orientations are labelled. **b-d,** TEM images and corresponding SAED patterns collected from the same color-coded viewing orientations as shown in **a** ($1\bar{1}0$, left; $01\bar{1}$, middle; $11\bar{1}$, right). Groove orientations and nanowire sidewall facets are labelled in individual panels.

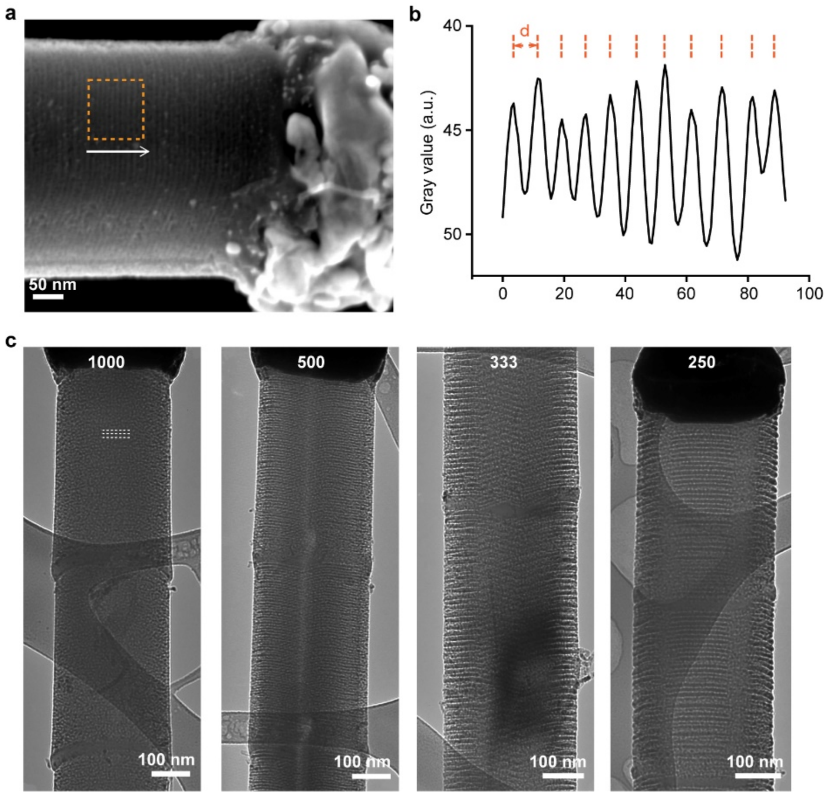

Supplementary Figure 9 | Spacing of grooves on Si nanowires. a, b, Statistical analysis of the groove spacing. **a,** A representative SEM image of an etched Si nanowire, showing prominent parallel grooves on its surface. The orange dashed box and the white arrow mark the region and the direction of analysis in **b**, respectively. **b,** A 1-D profile of the marked region highlights a periodic oscillation of the image grey value. Local minimal positions, marked by orange dashed lines, represent the centers of each groove. *d* represents the spacing value plotted in **Figure 4c**. **c,** TEM images of nanowires with different Si/P feeding ratios show the significant impact of doping levels on the groove formation. Intrinsic segments (smooth segments) were inserted in all cases to help visualize the interface shapes.

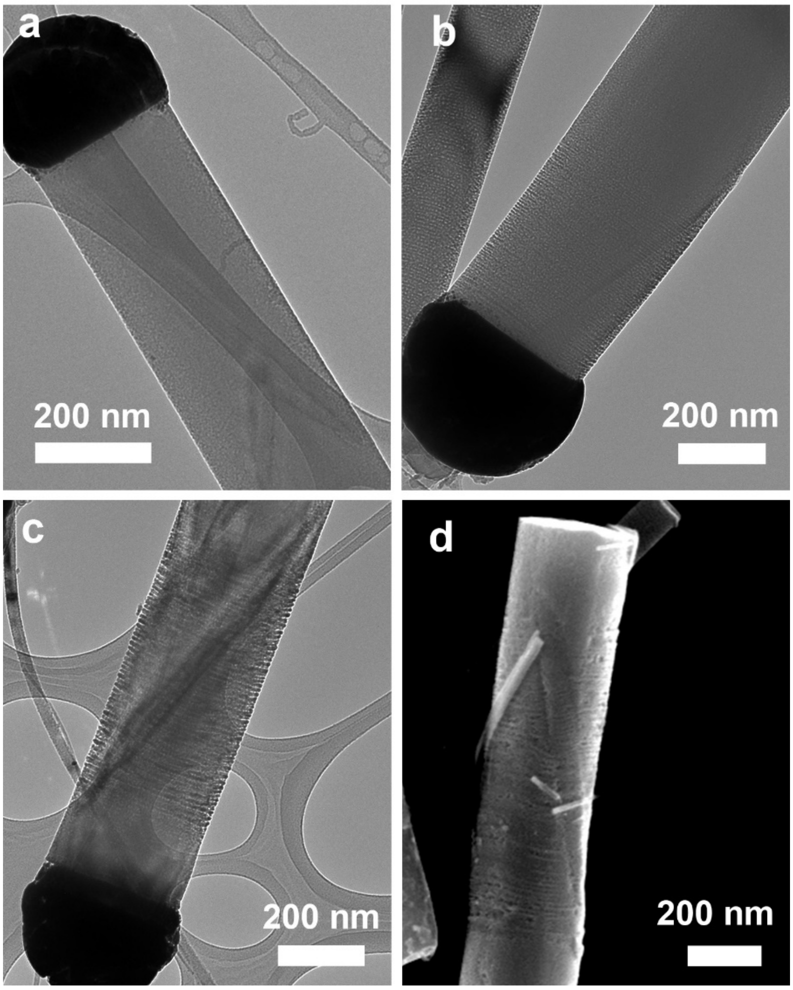

Supplementary Figure 10 | TEM and SEM images of etched Si nanowires with <111> growth orientation. a, The <111>-oriented nanowires do not show a high yield of parallel grooves after etching. **b-d**, The <111>-oriented nanowires show incoherent line patterns, and these lines are usually accumulated around the inclined {111} faults.

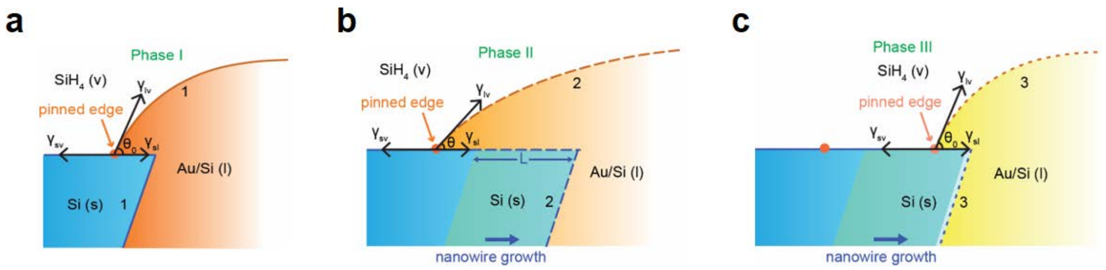

Supplementary Figure 11 | Three phases of SiNW growth (Phase I, II and III) during one cycle of stick-slip motion.

Supplementary Discussion

- **Analysis of atomic gold deposition.**

Overview

The formation of the parallel line pattern on silicon (Si) nanowires can be explained by a combination of a stick-slip motion that positions the liquid alloy droplet edge and a chemical potential variation that delivers Au.

Stick-slip motion

During the growth of an *n*-type Si nanowire, the dopant gas phosphine (PH_3) decomposes into phosphorus (P) which bonds strongly to Si[1-4]. Additionally, P also interacts robustly with Au species[5], leading to pinning of the droplet and an energy barrier for unpinning (U). During the nanowire growth, the alloy droplet contact angle is constantly decreasing because the triple-phase boundary (TPB) contact line is pinned at the equilibrium or quasi-equilibrium position. During the nanowire growth, the alloy droplet contact angle (θ) is initially decreasing because the TPB is pinned at the sidewall (i.e., 'Stick'). When θ reaches its minimum (θ_{min}), the potential barrier U can subsequently be overcome by the gain in Gibbs free energy (ΔG) upon snapping the TPB to the next equilibrium/quasi-equilibrium position with a contact angle θ_0 (i.e., 'Slip'). Because the Si chemical potential near the TPB is proportional to the contact angle, a reduction of θ upon nanowire elongation causes a local drop of $\mu_{Si,l}$, and correspondingly a local elevation of Au concentration and Au atom deposition near the TPB. However, unlike traditional droplet stick-slip motions due to either evaporation-induced droplet shrinking or directional droplet movement by external manipulation, the driving force for the current TPB dynamics is the elongation of Si nanowire through a VLS mechanism.

Detailed analysis of the stick-slip motion[6] can be performed based on an interfacial energy consideration. In most of the existing literature on VLS surface energetics, alloy droplet wetting over semiconductor sidewalls were not considered. However, our work revealed that this situation could occur. Specifically, when the TPB randomly shifts leftward and touches the solid sidewall, the droplet may get pinned if there is a strong 'retention' due to the presence of chemical species (in our case, phosphorus, P) over the semiconductor sidewalls (Phase I, **Supplementary Fig. 11a**).

At any given contact angle θ, the interfacial Gibbs free energy of the Au/Si alloy droplet can be described as

$$G = A_{lv}\gamma_{lv} + A_{sl}\gamma_{sl} - A_{sl}\gamma_{sv}, \qquad (1)$$

where A_{lv} and A_{sl} represent the contact areas of liquid/vapor and solid/liquid interfaces, γ_{lv}, γ_{sl} and γ_{sv} are the line tensions at liquid/vapor, solid/liquid and solid/vapor interfaces, respectively.

Under thermodynamic equilibrium, Young's contact angle equation gives

$$\gamma_{\mathrm{lv}} \cos\theta_0 = \gamma_{\mathrm{sv}} - \gamma_{\mathrm{sl}}\,, \tag{2}$$

where θ_0 is the equilibrium contact angle. Combining **Supplementary Eqs. 1** and **2**, the Gibbs free energy could be rewritten as

$$G = A_{\mathrm{lv}}\gamma_{\mathrm{lv}} - A_{\mathrm{sl}}\gamma_{\mathrm{lv}}\cos\theta_0\,. \tag{3}$$

During nanowire growth, the TPB contact line is assumed to become pinned at its equilibrium/quasi-equilibrium position starting from a certain time point. When the nanowire continues to elongate for an extra length of L, this newly grown segment would cause an increase of the solid/liquid interfacial area by $L\times d$, where d is a geometrical factor at the TPB. The corresponding contact angle of the alloy droplet decreases from θ_0 to θ (Phase II, **Supplementary Fig. 11b**).

The excess Gibbs free energy under the non-equilibrium state is

$$\begin{aligned}\Delta G = G - G_0 &= (A_{\mathrm{lv}} - A_{\mathrm{lv}}^0)\gamma_{\mathrm{lv}} - (A_{\mathrm{sl}} - A_{\mathrm{sl}}^0)\gamma_{\mathrm{lv}}\cos\theta_0 \\ &= (A_{\mathrm{lv}} - A_{\mathrm{lv}}^0)\gamma_{\mathrm{lv}} - Ld\gamma_{\mathrm{lv}}\cos\theta_0\end{aligned}\,. \tag{4}$$

Besides θ, A_{lv} is also a function of d from the geometrical analysis of the alloy droplet shape.

When the excess Gibbs free energy reaches its maximum.

$$\Delta G_{\mathbf{max}} = U\,, \tag{5}$$

where U is the pinning energy due to the interaction between Au/Si liquid alloy and solid n-type Si nanowire sidewall, the droplet has enough driving force to overcome the pinning potential barrier so the TPB contact line can jump to its next equilibrium/quasi-equilibrium position (Phase III, **Supplementary Fig. 11c**)[7]. In general, U should be highly dependent on the chemical environment during the VLS growth. In our case, the higher the PH_3 feeding concentration, the higher the amplitude of U as it is related to the interaction between P and Au/Si.

Chemical potential variation

During the above stick-slip motion, Au atoms would be deposited on the Si nanowire sidewall due to contact-angle-dependent Au delivery. This process can be understood by a chemical potential argument.

In a typical Si nanowire growth process, the Gibbs free energy change during the process of Si deposition at TPB can be described as[8]

$$\Delta G_{\mathrm{Si}} = G_{\mathrm{Si,s}} - G_{\mathrm{Si,l}} = (\mu_{\mathrm{Si,s}} - \mu_{\mathrm{Si,l}} + \delta\sin\theta)N_{\mathrm{Si}}\,, \tag{6}$$

where $\mu_{Si,s}$ and $\mu_{Si,l}$ are the chemical potentials of Si in the solid nanowire and liquid alloy, respectively, δ is a positive constant that is relevant to the geometry and energy at TPB and displaces a property of a *δ-function*, θ is the alloy droplet contact angle, N_{Si} is the total number of Si under consideration. The contact angle dependence is introduced due to a force normal to the solid-liquid interface that induced a stress on the nanowire sidewall[8].

Since the nanowire has a uniform diameter, *i.e.*, there is no radial deposition of Si atoms, therefore $\Delta G_{Si}=0$. **Supplementary Eq. 6** can be reorganized to[8]

$$\mu_{Si,l} - \mu_{Si,s} = \delta \sin\theta . \qquad (7)$$

Within a single oscillation cycle, the contact angle is constantly decreasing from the initial pinning time point, due to the stretching of the liquid alloy droplet by the newly elongated segment. From **Supplementary Eq. 7**, the chemical potential difference of Si $(\mu_{Si,s} - \mu_{Si,l})$ at TPB reaches its maximum at the minimum contact angle, which triggers Au deposition at the solid Si nanowire surface to counterbalance the change of $(\mu_{Si,s} - \mu_{Si,l})$. The extraction of Au atoms from the alloy droplet and their deposition at the TPB may also be facilitated by the strong interaction between P and Au. Finally, since such a chemical potential variation follows a *δ-function* around the TPB, we therefore only expect Au deposition in the form of lines, instead of patches.

Supplementary Methods

- **Statistical analysis of groove spacing**

Overview

An ***R*** project was applied for the statistical analysis of SEM images. The code used for the computing process in ***R*** is shown below. We ran the code step-by-step with instructions for each part shown in the comment lines (marked with ##).

Code for statistical analysis:

```
```{r}
setwd("D:/ Data/sample") ## set working directory

data<-read.table("D:/ Data/sample/sample 1.txt", header=TRUE)
plot(data$X, data$Y, type='l') ## read in data file and plot data

maximums <- function (x, m = 5){
        shape <- diff(sign(diff(x)))
        pks <- sapply(which(shape < 0), FUN = function(i){
              z <- i - m + 1
              z <- ifelse(z > 0, z, 1)
              w <- i + m + 1
              w <- ifelse(w < length(x), w, length(x))
              if(all(x[c(z : i, (i + 1) : w)] <= x[i + 1])) return(i) else
return(numeric(0))
        })
          pks <- unlist(pks)
          pks + 1
}
maximum<-maximums(data$Y) ## determine and save maxima

length<-length(maximum)
maxima<-rep(0,length)
for(i in 1:length){
    maxima[i]<-data$X[maximum[i]]
}    ## initialize and populate the maxima x-value vector (does not include initial, non
maximum point). Used to save maxima.csv

maximasplit<-rep(data$X[1],length+1)
for(i in 1:length){
maximasplit[i+1]<-data$X[maximum[i]]
```
```

```
} ## initialize and populate maxima x-value vector for splitting data set (includes potential
non-maximum point, i.e. the first x-value in the data set)

data$Y<-data$Y*-1 ## make intensity values negative to invert curves

df<-split(data, cut(data$X, breaks = maximasplit, include.lowest = TRUE, labels = FALSE))
## split data set by maxima x-values
dir <- getwd()
lapply(seq_len(length(df)),
        function (i) write.csv(df[[i]], file = paste0(dir,"/file",names(df[i]), ".csv"), row.names
= FALSE)) ## save split data set as .csv files

minimums <- function (x, m = 5){
    shape <- diff(sign(diff(x)))
    pks <- sapply(which(shape < 0), FUN = function(i){
        z <- i - m + 1
        z <- ifelse(z > 0, z, 1)
        w <- i + m + 1
        w <- ifelse(w < length(x), w, length(x))
        if(all(x[c(z : i, (i + 1) : w)] <= x[i + 1])) return(i) else
return(numeric(0))
    })
     pks <- unlist(pks)
     pks + 1
}
minimum<-minimums(data$Y) ## determine and save minima

minlength<-length(minimum)
minima<-rep(0,minlength)
for(i in 1:minlength){
  minima[i]<-data$X[minimum[i]]
} ## initialize and populate minima x-value vector

mindif<-rep(0,minlength-1)
for(i in 1:minlength){
  mindif[i]<-minima[i+1]-minima[i]
} ## initialize and populate minima x-value distance/difference vector

write(maxima, file = "maxima.csv",
      ncolumns = 1,
      append = FALSE, sep = ", ") ## save maxima x-values as csv data file

write(minima, file = "minima.csv",
      ncolumns = 1,
```

```
    append = FALSE, sep = ", ") ## save minima x-values as csv data file

write(mindif, file = "mindif.csv",
    ncolumns = 1,
    append = FALSE, sep = ",") ## save minima distance values as csv data file
```

Supplementary References:

1. Connell, J. G. *et al.* Identification of an intrinsic source of doping inhomogeneity in vapor-liquid-solid-grown nanowires. *Nano Lett.* **13**, 199-206, (2013).
2. Perea, D. E. *et al.* Direct measurement of dopant distribution in an individual vapour-liquid-solid nanowire. *Nat. Nanotechnol.* **4**, 315-319, (2009).
3. Xie, P., Hu, Y. J., Fang, Y., Huang, J. L. & Lieber, C. M. Diameter-dependent dopant location in silicon and germanium nanowires. *Proc. Natl. Acad. Sci. U. S. A.* **106**, 15254-15258, (2009).
4. Yu, M. L., Vitkavage, D. J. & Meyerson, B. S. Doping reaction of PH_3 and B_2H_6 with Si(100). *J. Appl. Phys.* **59**, 4032-4037, (1986).
5. Schmid, H. *et al.* Doping limits of grown in situ doped silicon nanowires using phosphine. *Nano Lett.* **9**, 173-177, (2009).
6. Shanahan, M. E. R. Simple theory of stick-slip wetting hysteresis. *Langmuir* **11**, 1041-1043, (1995).
7. Oksuz, M. & Erbil, H. Y. Comments on the energy barrier calculations during "Stick-Slip" behavior of evaporating droplets containing nanoparticles. *J. Phys. Chem. C* **118**, 9228-9238, (2014).
8. Gamalski, A. D., Ducati, C. & Hofmann, S. Cyclic supersaturation and triple phase boundary dynamics in germanium nanowire growth. *J. Phys. Chem. C* **115**, 4413-4417, (2011).
