# Supplementary material for: Alloy-assisted deposition of three-dimensional arrays of atomic gold catalyst for crystal growth studies
Source: Nat Commun. 2017 Dec 8;8:2014. doi: 10.1038/s41467-017-02025-x (PMC5722855; doi:10.1038/s41467-017-02025-x)
Supplement: Supplementary file 2 — Description of Additional Supplementary Information [file 41467_2017_2025_MOESM2_ESM.pdf]

Description of Additional Supplementary Files

File Name: Supplementary Movie 1
Description: *Ab initio* molecular dynamics (AIMD) simulation of a model Si(111) surface with an isolated surface Au atom. An Au atom was placed on a Si site in the initial structure.10 molecules each of HF and H_2O_2 were placed randomly on the (111) surface of Si. Total duration: 50 ps.

File Name: Supplementary Movie 2
Description: *Ab initio* molecular dynamics (AIMD) simulation of a model Si(111) surface. 10 molecules each of HF and H_2O_2 were placed randomly on the (111) surface of Si. Total duration: 50 ps.
